# Effects of Simulated Precipitation Treatment on Denitrifying Microbial Communities in the Wayan Mountains

**DOI:** 10.3390/biology15060512

**Published:** 2026-03-23

**Authors:** Shijia Zhou, Kelong Chen, Ni Zhang, Zhiyun Zhou, Siyu Wang

**Affiliations:** 1 Qinghai Province Key Laboratory of Physical Geography and Environmental Process, College of Geographical Science, Qinghai Normal University, Xining 810008, China; ruye1125@163.com (S.Z.); 13897423633@163.com (Z.Z.); 15229584811@163.com (S.W.); 2 Key Laboratory of the Qinghai–Tibet Plateau Land Surface Processes and Ecological Conservation (Ministry of Education), Qinghai Normal University, Xining 810008, China; 3 National Positioning Observation and Research Station of Qinghai Lake Wetland Ecosystem in Qinghai, National Forestry and Grassland Administration, Haibei 812300, China

**Keywords:** Qinghai–Tibet Plateau, alpine wetlands, precipitation variation, *nirK* gene, denitrifying microorganisms

## Abstract

The Qinghai–Tibet Plateau is undergoing rapid warming and humidification, with altered precipitation regimes increasingly affecting soil nitrogen cycling and N_2_O emissions. Denitrification—a key nitrogen transformation pathway—is particularly sensitive to these hydrological changes. Here, we investigated the response of *nirK*-type denitrifying microbial communities to altered precipitation in an alpine wetland on the northern shore of Qinghai Lake. Using a long-term precipitation manipulation platform with five gradients (ambient, ±25%, and ±50%), we integrated high-throughput sequencing with bioinformatics to systematically assess community shifts. Short-term precipitation treatments did not significantly alter alpha diversity, but markedly restructured community composition. Extreme wetting (+50%) increased within-group heterogeneity. At the phylum level, Proteobacteria remained dominant across all treatments, whereas extreme drought (−50%) suppressed Planctomycetes. At the genus level, *Ochrobactrum* was enriched under reduced precipitation, while *Rhodopseudomonas* increased under increased precipitation. Functional predictions indicated that reduced precipitation enhanced nitrogen fixation potential, whereas increased precipitation favored nitrate respiration. Soil pH and carbon fractions were the key environmental filters driving community variation. Ecological process analysis revealed that community assembly was entirely governed by deterministic processes, specifically variable selection. Together, these findings elucidate how precipitation shifts reconfigure the structure and functional potential of denitrifying microbial communities in alpine wetlands, primarily via changes in soil pH and moisture under variable selection. This work provides critical insights into microbial regulation of the nitrogen cycle on the Tibetan Plateau under ongoing climate change.

## Simple Summary

Precipitation regimes on the Qinghai–Tibet Plateau are shifting, yet how these changes affect denitrifying microbes—key regulators of soil nitrogen cycling—remains poorly understood. Through a simulated precipitation experiment in the Wayan Mountain alpine wetland, we show that although short-term precipitation alterations (±25%, ±50%) did not affect the alpha diversity of *nirK*-type denitrifying communities, they profoundly reshaped community composition and functional potential. Reduced precipitation enriched *Ochrobactrum* and enhanced nitrogen fixation potential, whereas increased precipitation favored *Rhodopseudomonas* and strengthened nitrate respiration. Extreme drought suppressed Planctomycetes. Soil pH emerged as the dominant environmental driver, and community assembly was governed entirely by deterministic processes (variable selection). These findings reveal how precipitation modulates denitrifier communities via soil filtering, providing mechanistic insight into nitrogen cycle responses to climate change on the Tibetan Plateau.

## 1. Introduction

Nitrous oxide (N_2_O) is the third most potent greenhouse gas, with a global warming potential ~273 times that of CO_2_ over a 100-year horizon [1]. The Qinghai–Tibet Plateau—the “Asian Water Tower”—has warmed at nearly twice the global average rate in recent decades, accompanied by regionally increased precipitation. This warming and wetting trend has intensified regional hydrological cycling, thereby modulating soil nitrogen transformation and greenhouse gas emissions [2]. Precipitation shifts can substantially alter N_2_O emissions, but the direction and magnitude of these effects vary across ecosystems. In alpine meadows of the Qinghai–Tibet Plateau, extreme precipitation changes enhanced N_2_O emissions by altering microbial community composition and functional gene abundance [3]. In the same system, nitrogen addition alone increased N_2_O emissions by 68.8%, increased precipitation alone by 53.9%, and their interaction by 44.5%—suggesting that wetting may attenuate N_2_O fluxes under elevated nitrogen deposition [4]. Conversely, a precipitation manipulation experiment (−70% to +50%) in Northeast China reported that growing-season N_2_O fluxes declined with decreasing precipitation and were positively correlated with soil moisture [5]. In a desert steppe of Northwest China, however, increased precipitation (40% and 60%) elevated N_2_O emissions only in the early growing season, with no significant annual effect [6]. In a semi-arid grassland, moderate precipitation reduction unexpectedly stimulated nitrogen cycling and increased N_2_O emissions, contrasting with the inhibitory effects typically associated with extreme drought [7]. This nonlinearity underscores the complexity of precipitation–N_2_O relationships. In agricultural systems, nitrogen fertilizer and soil moisture have been identified as key drivers of N_2_O release [8].

Denitrification is a central process in the soil nitrogen cycle, functioning as a self-regulatory mechanism that prevents nitrogen imbalance. The sequential reduction of NO_3_^−^ to N_2_ is catalyzed by four reductases; nitrite reductase (encoded by *nirK* and *nirS*) catalyzes the rate-limiting step [9,10]. The *nirK* gene is prevalent across phylogenetically diverse bacteria and is generally more sensitive to environmental change than *nirS*, making it a robust indicator of ecosystem perturbation [11,12]. As early as 1997, researchers systematically elucidated the biochemical and genetic foundations of denitrification, establishing a research paradigm centered on model strains such as *Pseudomonas* and *Paracoccus*, which provided molecular benchmarks for subsequent studies on community function [13]. Laurent Philippot [14] expanded the research scope from bacteria to archaea, employing genomic analyses to reveal the distribution and evolution of denitrification genes across the microbial world, thereby advancing the concept that “the diversity of denitrifying microorganisms far exceeds previous expectations.” Braker et al. [15]. developed molecular detection tools targeting key functional genes (*nirK*, *nirS*), transforming culture-independent techniques into the mainstream approach for studying denitrifying communities in environmental samples—a pivotal technological breakthrough that shifted the conceptual framework from pure culture to environmental community research. Numerous studies have demonstrated [16,17] that, by identifying indicator microbial taxa sensitive to environmental factors such as moisture, pH, and temperature, researchers can detect early warnings of soil functional degradation or nitrogen cycle imbalance through shifts in microbial community structure [18]. This enables the implementation of interventions before the loss of ecosystem services occurs. Using *nirS* and *nirK* as markers, Niu et al. [19] showed that soil type, vegetation, and seasonality jointly shape denitrifier community structure in salt marsh wetlands. Other work has reported positive correlations between *nirK* abundance and soil moisture, implying that wetter conditions favor denitrifier proliferation [20]. Moreover, the combined abundance of *nirK* and *nirS* genes is closely linked to precipitation regimes and influences net nitrification and N_2_O emissions [21]. Given projected increases in precipitation over the Qinghai–Tibet Plateau, the response of soil denitrification to altered hydrology demands urgent investigation.

The water resources, mountain ranges, and alpine meadows of the Qinghai Lake Basin form a critical ecological barrier that prevents desertification from expanding eastward [22]. Climate change is expected to further alter precipitation regimes across the Asian Water Tower [23], with cascading effects on soil carbon and nitrogen turnover [24]. Yet the response of *nirK*-type denitrifying communities to precipitation variability in this basin remains poorly understood. To address this gap, we conducted a precipitation manipulation experiment in the Wayan Mountain alpine wetland—a representative ecosystem on the northeastern Qinghai–Tibet Plateau Using high-throughput sequencing targeting the *nirK* gene, we investigated how long-term precipitation changes and key environmental variables shape the community composition and functional potential of *nirK*-type denitrifiers. This study directly elucidates the relationship between denitrifying microbial communities and N_2_O emissions; it also supports the feasibility of using microorganisms as early warning indicators for the Qinghai Lake watershed ecosystem. Specifically, we asked: (1) Does species richness and alpha diversity of *nirK*-type denitrifiers correlate with precipitation amount? (2) To what extent do different precipitation levels influence community complexity and stability?

## 2. Materials and Methods

### 2.1. Overview of the Study Area

**Figure 1:**
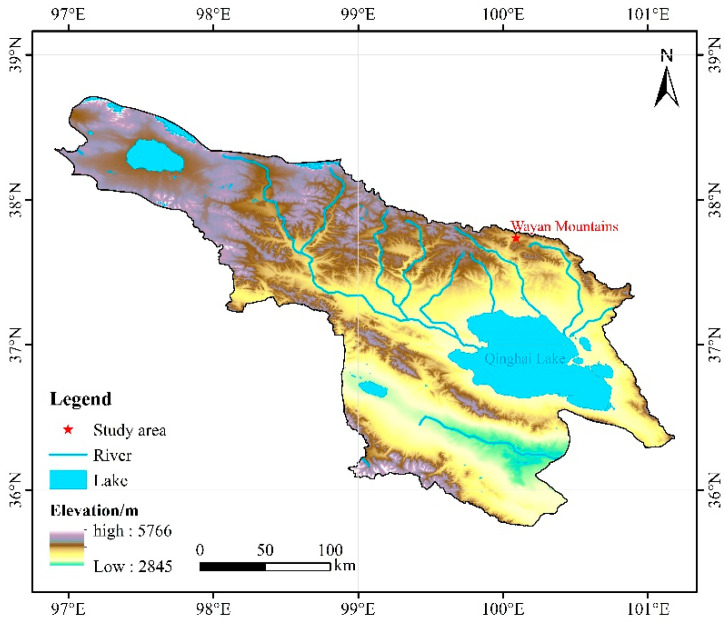
Study area overview map.

Around 2004/2005, the water level of Qinghai Lake underwent a distinct reversal, transitioning from a prolonged decline to a continuous rise, with an average annual increase of 0.21–0.30 m. This rise is primarily attributable to increased precipitation driven by climate change and enhanced glacial and snowmelt runoff, and it has significantly affected the lake’s surface area, water storage, coastal ecosystems, and regional hydrogeological dynamics. As such, Qinghai Lake serves as a key location for studying the coupled interactions among climate, hydrology, and ecology on the Tibetan Plateau [25,26]. The Qinghai Lake basin, located in the northeastern part of the plateau, functions as an essential ecological barrier for regional environmental security. The present study was conducted at the Wayan Mountain Experimental Station, situated on the northeastern shore of the lake, within the geographic coordinates 37°43′–37°46′ N and 100°01′–100°05′ E (Figure 1). Field experiments employed natural precipitation as a baseline, with precipitation amounts modified by ±25% and ±50%. Reduced precipitation was achieved by installing evenly spaced inclined water channels that diverted rainwater into horizontal reservoirs within convergence channels. Increased precipitation was implemented via spray systems that supplemented natural rainfall. The treatments were designated as follows: Wck (ambient control), ZA (+50% precipitation), ZB (+25% precipitation), JA (−50% precipitation), and JB (−25% precipitation). Each treatment was replicated in three plots.

### 2.2. Sample Collection and Physicochemical Determination

In June 2022, topsoil samples (0–10 cm depth) were collected using a 4.5 cm-diameter auger. Within each plot, five sampling points were established following a five-point pattern, and the five cores were combined to form a composite sample. After collection, the soil was passed through a 2 mm sieve to remove plant roots, stones, and other coarse materials. Samples were immediately transported to the laboratory in insulated containers with ice packs. Subsamples intended for physicochemical analyses were stored at −4 °C, while those for molecular analyses were preserved at −80 °C.

The TDR-300 Soil Moisture Probe (Spectrum Technologies Inc., Plainfield, IL, USA) monitored soil moisture content. LI-8100 system (LI-COR, Lincoln, NE, USA), respectively. Soil pH was determined using a pH probe (FE20-FiveEasy pH, Mettler Toledo, Giessen, Germany) with a soil–water ratio of 1:2.5. The contents of total carbon (TC) and total nitrogen (TN) were determined using the Elemental Analysis System GmbH (Vario EL III, Frankfurt, Germany) [27].

### 2.3. DNA Extraction and Illumina MiSeq Sequencing

Total DNA was extracted from 0.5 g of soil using the PowerSoil DNA Isolation Kit (Mo Bio, Carlsbad, CA, USA), and DNA integrity was verified by electrophoresis on a 1% agarose gel. The *nirK* gene fragment was amplified with primers copper583F (5′-ATC ACC AGG GAC GTC GGC GAK-3′) and copper909R (5′-GAC CTT GGT GGT TTR AAG TG-3′). PCR products were purified and subjected to paired-end sequencing on an Illumina MiSeq platform (Illumina, San Diego, CA, USA). Raw reads were demultiplexed and primer sequences were trimmed using Cutadapt (v1.9.1). Quality filtering, denoising, and inference of amplicon sequence variants (ASVs) were performed with Usearch (v10.0) [28] and the DADA2 pipeline implemented in QIIME2 (v2020.6) [29]. Taxonomic assignment of ASVs was carried out by comparison against the NCBI Non-Redundant Protein Database (NR).

### 2.4. Statistical Analysis

All statistical analyses were conducted in R (v4.1.2). Alpha diversity indices (e.g., Chao1, Shannon, Simpson) were calculated using the MicrobiotaProcess package (v4.3.2). The distribution of ASVs among samples was visualized with UpSetR (v1.4.0). Beta diversity was assessed via principal component analysis (PCA) with the PCAtools package (v2.14.0) and non-metric multidimensional scaling (NMDS) using vegan (v2.6.6.1) based on Bray–Curtis distances. Permutational multivariate analysis of variance (PERMANOVA, Adonis) and analysis of similarities (ANOSIM) were applied to test for significant differences in community composition. Redundancy analysis (RDA) was also performed with vegan to relate community structure to environmental variables. Correlations between variables were computed with the psych package (v2.4.3), and significance was determined via ANOVA (aov function from the stats package). Functional profiles of denitrifying microorganisms were predicted using FAPROTAX [30]. Association networks and correlation heatmaps were generated with the linkET package (v0.0.7.4) and visualized with qcorrplot, while additional heatmaps were produced with pheatmap (v1.0.12). Structural equation modeling (SEM) was carried out with piecewiseSEM (v2.3.0) to examine direct and indirect effects of environmental factors on the diversity and composition of *nirK*-type denitrifiers. To quantify the relative importance of deterministic versus stochastic processes in community assembly, the β-nearest taxon index (βNTI) was calculated using a null model with the picante package (v1.8.2), and the Raup–Crick metric (RCbray) was computed with the Microecology package (v1.7.1). The study area map was created with ArcGIS (v10.8).

## 3. Results

### 3.1. Response of nirK-Type Denitrifying Bacterial Community Diversity to Precipitation Variation

**Figure 2:**
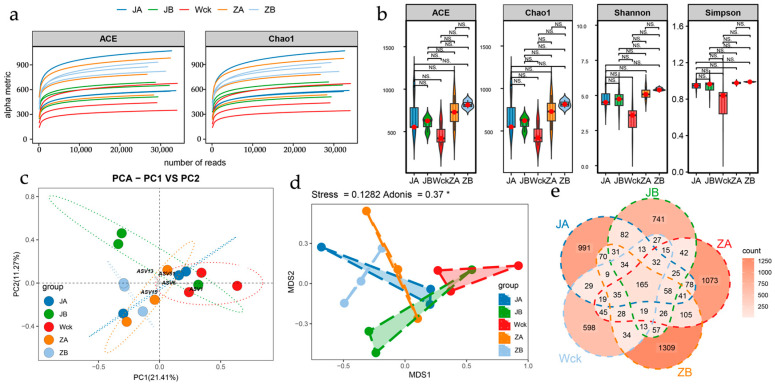
*NirK* sequencing results after simulated precipitation treatment; (**a**) sample dilution curve; (**b**) α Diversity Index of Denitrifying Microorganisms. (**c**) Principal Component Analysis of *nirK* Denitrifying Microorganisms Following Simulated Precipitation Treatments; (**d**) Analysis of differences between groups; (**e**) Shared and Unique Patterns of nirK-Type Denitrifying Microbial OTUs Under Different Precipitation Treatments. Wck: natural contrast; ZA: 50% increase in rainfall; ZB: 25% increase in rainfall; JA: 50% decrease in rainfall; JB: 25% decrease in rainfall; NS: No significant; *: *p* < 0.05.

At sequencing depths ranging from 20,000 to 30,000 reads, the rarefaction curves of both the ACE and Chao1 indices for all samples reached a clear plateau, indicating that the current sequencing depth was sufficient to capture the vast majority of species within each sample (Figure 2a). Compared with the control group (Wck), none of the alpha diversity indices of soil *nirK*-type denitrifying microbial communities exhibited statistically significant changes under any of the precipitation treatments (JA, JB, ZA, ZB; *p* > 0.05). Although the richness estimators (ACE and Chao1 indices) were marginally higher in the ZB treatment group relative to the other groups, inter-group differences were not significant. Likewise, no significant differences were detected in the Shannon and Simpson indices across treatments; notably, the Simpson index displayed an increasing trend in the ZB group, but this trend did not attain statistical significance (Figure 2b). Collectively, these results suggest that short-term precipitation alterations did not markedly affect species richness or the overall diversity structure of the target microbial community. For details on alpha diversity, see Table S1 in the Supplementary Materials.

Principal component analysis (PCA) revealed that differential precipitation regimes exerted a substantial impact on the community structure of *nirK*-type denitrifiers. Precipitation reduction treatments were clearly separated from both the control and precipitation enhancement treatments along the principal axes, implicating reduced precipitation as a key driver of shifts in community composition. Moreover, the extreme precipitation enhancement treatment (ZA) exhibited the highest within-group dispersion, suggesting that such treatment may induce more stochastic and unpredictable community responses (Figure 2c). In line with this, multidimensional scaling (MDS) analysis corroborated that precipitation treatments significantly altered the community structure of *nirK*-harboring denitrifiers (Adonis: R^2^ = 0.37, *p* < 0.05). Specifically, the reduced precipitation treatments (JA and JB) promoted community homogenization, whereas the extreme increased precipitation treatment (ZA) significantly elevated intra-group heterogeneity (Figure 2d).

Venn diagram analysis at the operational taxonomic unit (OTU) level elucidated the structuring effects of precipitation treatments on *nirK*-type denitrifying communities (Figure 2e). A total of 13 core OTUs were shared across all treatment groups, indicating the presence of a stable functional guild resilient to precipitation variations. Notably, the extreme treatments (JA and ZA) harbored the highest numbers of unique OTUs (25 and 32, respectively), confirming that severe hydrological perturbations drive the emergence of distinctive taxonomic compositions. Furthermore, the two increased precipitation treatments (ZA and ZB) shared the largest number of OTUs (70), substantially exceeding the shared counts observed between any other treatment pairs.

### 3.2. Response of Denitrifying Microbial Species Composition and Functional Groups to Precipitation Variability

**Figure 3:**
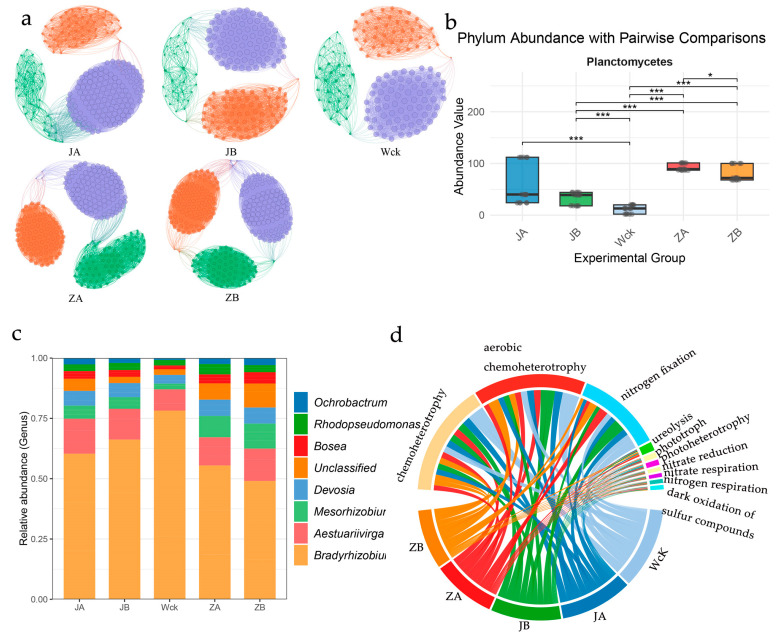
Response of Species Composition and Functional Groups in denitrifying microbial communities to simulated precipitation variations. (**a**) Co-occurrence Network Diagram. Orange module: Core stability module; Purple module: Auxiliary stability module; Green module: Precipitation response module (**b**) Phylum Abundance with Pairwise Comparisons. (**c**) Composition of genus-level communities. (**d**) Relative abundance and composition of major functional groups. Wck: natural contrast; ZA: 50% increase in rainfall; ZB: 25% increase in rainfall; JA: 50% decrease in rainfall; JB: 25% decrease in rainfall; *: *p* < 0.05; ***: *p* < 0.001.

Microbial co-occurrence network analysis revealed that, under the control and mild precipitation treatments, the *nirK*-type denitrifying community exhibited a complex network topology characterized by dense connectivity and well-defined modular structure, indicative of stable inter-species cooperation within the community. In contrast, extreme precipitation reduction (JA) resulted in pronounced network simplification and diminished connectivity, reflecting niche compression under drought stress. Extreme precipitation enhancement (ZA), on the other hand, triggered substantial network reorganization, manifested by disrupted modular architecture and altered connectivity patterns—findings that are consistent with the elevated within-group heterogeneity observed in the beta diversity analyses (Figure 3a).

In-depth taxonomic profiling of *nirK*-type denitrifying communities across precipitation treatments demonstrated that, at the phylum level, Proteobacteria remained overwhelmingly dominant across all treatments (relative abundance > 95%), indicating that the overarching compositional architecture of this functional microbial guild at the phylum level was not substantively altered by precipitation manipulation. Nevertheless, further phylum-level interrogation identified specific taxa sensitive to precipitation shifts. Although the overall dominance structure remained stable, the phylum Planctomycetes exhibited significant and specific suppression under extreme precipitation reduction (JA) (Figure 3b). This finding underscores the potential utility of Planctomycetes as a bioindicator of arid conditions; its marked decline represents one of the most definitive microbial signals in response to extreme water deficit. Genus-level compositional analysis further elucidated the specific effects of precipitation regimes on *nirK*-type denitrifying communities. The dominant genus *Bradyrhizobium* maintained its predominance across all treatments (relative abundance > 50%). Notably, however, the relative abundance of *Ochrobactrum* increased significantly under reduced precipitation, peaking in the JA treatment, whereas *Rhodopseudomonas* exhibited a distinctly higher proportional representation under increased precipitation treatments (ZA, ZB) (Figure 3c).

Functional predictions based on FAPROTAX further illuminated the profound influence of precipitation variability on microbial ecological functions. Sankey diagram analysis illustrated a redistribution of core metabolic functions—such as aerobic chemoheterotrophy—across treatments. Critically, nitrogen-cycling functions displayed marked sensitivity to precipitation regimes: nitrogen_fixation exhibited a relatively enhanced trend under reduced precipitation, particularly in the JA treatment; conversely, the functional potential for nitrate_respiration was notably amplified under increased precipitation, especially in the ZA treatment. These functional shifts provide robust evidence at the metabolic level that precipitation reduction may steer the microbial community toward a more conservative nitrogen retention strategy, whereas precipitation increase potentially exacerbates nitrogen loss via denitrification-dominated pathways. This functional interpretation is highly congruent with the observed responses of the *nirK*-type denitrifying community in the present study (Figure 3d). For details on the functional analysis, see Supplementary Material Table S3.

### 3.3. Correlation Between Environmental Factor Variations and the nirK Denitrifying Microbial Community

**Figure 4:**
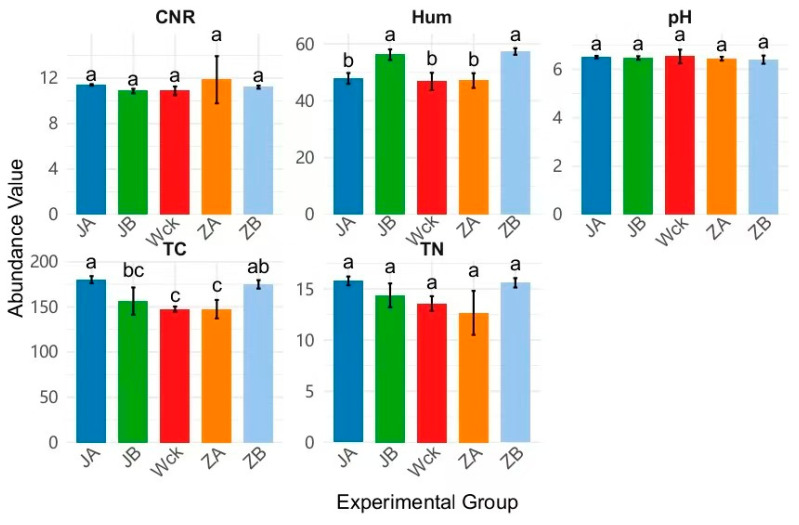
Changes in Soil Physicochemical Properties Across Different Precipitation Gradients. CNR: Carbon to Nitrogen Ratio, Hum: Soil humus, TN: total nitrogen, TC: total carbon, pH: soil pH. Wck: natural contrast; ZA: 50% increase in rainfall; ZB: 25% increase in rainfall; JA: 50% decrease in rainfall; JB: 25% decrease in rainfall. Differences between groups labeled with the same letter above the bar chart are not significant (*p* > 0.05), while differences between groups labeled with different letters are significant (*p* < 0.05).

**Figure 5:**
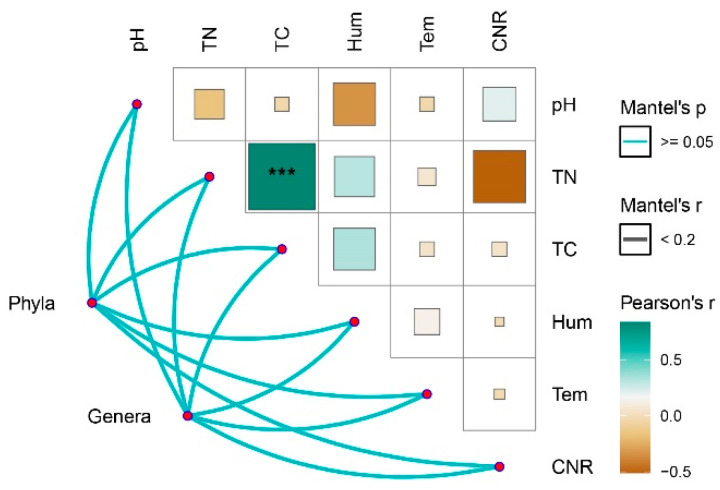
Correlation Network Diagram of *nirK* Denitrifying Microbial Community Characteristics and Environmental Factors. CNR: Carbon to Nitrogen Ratio, Hum: Soil humus, TN: total nitrogen, TC: total carbon, pH: soil pH, Tem: soil temperature. Wck: natural contrast; ZA: 50% increase in rainfall; ZB: 25% increase in rainfall; JA: 50% decrease in rainfall; JB: 25% decrease in rainfall; JA: 50% decrease in rainfall; JB: 25% decrease in rainfall; ***: *p* < 0.001.

**Figure 6:**
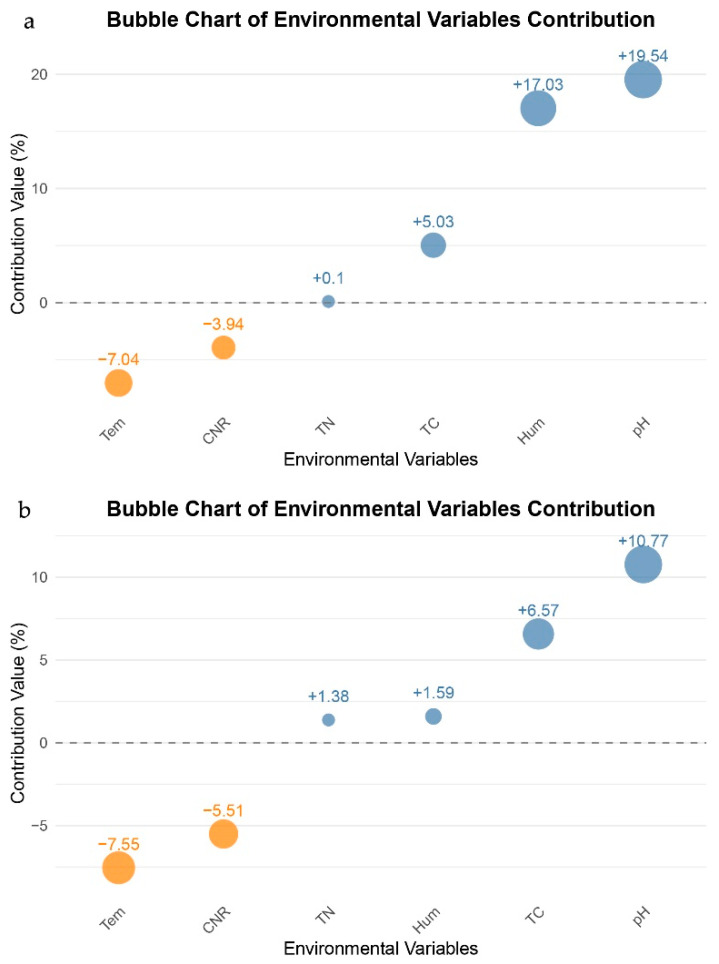
(**a**) Contribution of Environmental Variables to Microbial Community Diversity. (**b**) Environmental Variables Contributing to Microbial Community Structure. Blue bubbles: indicate a positive contribution. Orange bubbles: indicate a negative contribution. Dotted lines: indicate positive and negative contributions.

Analysis of soil physicochemical properties revealed that precipitation treatments exerted significant effects on key environmental variables. Specifically, the JA (extreme drought) treatment led to significant increases in CNR (Carbon to Nitrogen Ratio) and total carbon content, while the ZA extreme wetting treatment significantly elevated Hum (Soil humus) (Figure 4). In contrast, no significant differences were detected in pH or TN across any of the treatments. Correlation network analysis further demonstrated a highly significant positive association between TN and TC. However, *nirK*-type denitrifying communities were not significantly affected by these environmental factors at either the phylum or genus level (Figure 5). Further contribution analysis of environmental variables to microbial community diversity indicated that pH (19.54%) and Hum (17.03%) were the two predominant drivers of diversity differentiation, whereas TC exhibited only a modest positive explanatory power (5.03%). Notably, temperature (Tem) contributed the largest negative effect (−7.04%), while TN displayed a negligible contribution (0.1%) (Figure 6a). With respect to microbial community structure, the explanatory power of individual environmental factors varied markedly. pH (10.77%) and TC (+6.57%) emerged as the two most influential positive drivers of structural differentiation. Conversely, Tem (−7.55%) and CNR (−5.51%) exerted substantial negative contributions. In contrast, the contributions of Hum and TN were minimal (both < 1.6%) (Figure 6b). Details of soil physical and chemical factors are shown in Supplementary Materials Table S2.

### 3.4. Assembly Process of nirK Denitrifying Microbial Communities Under Precipitation Variations

**Figure 7:**
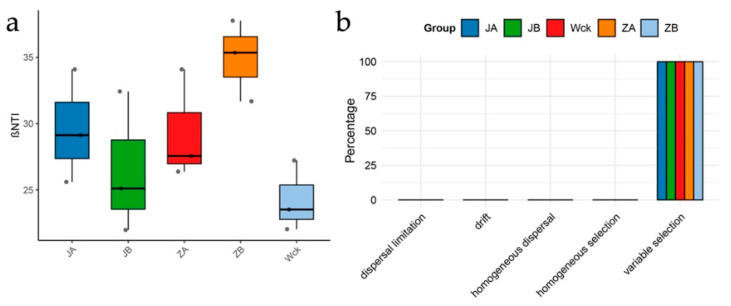
(**a**) BetaNTI Index for Different Groups. (**b**) Distribution of community construction processes across different groups. Wck: natural contrast; ZA: 50% increase in rainfall; ZB: 25% increase in rainfall; JA: 50% decrease in rainfall; JB: 25% decrease in rainfall.

To elucidate the ecological processes governing shifts in microbial community composition, we conducted beta nearest taxon index (βNTI) analysis. The results revealed that βNTI values across all precipitation treatment groups were significantly greater than +2 (Figure 7a), confirming that deterministic processes overwhelmingly dominated community assembly. Further partitioning of assembly processes demonstrated that “variable selection” accounted for a relative contribution approaching 100% in each of the treatment groups—JA, JB, ZA, ZB, and the control (Wck) (Figure 7b). This provides compelling evidence that heterogeneity in soil environmental conditions, induced by divergent precipitation regimes, constitutes the fundamental ecological driver shaping and differentiating the community structure of *nirK*-type denitrifying community.

## 4. Discussion

### 4.1. Alpha Diversity Is Insensitive to Short-Term Precipitation Change

Soil microbial communities often respond to precipitation shifts [31]. A substantial body of evidence supports this contention. For instance, Yu et al. [32] demonstrated in a precipitation manipulation experiment that soil microbial biomass increased concomitantly with elevated precipitation, and this response was reversible upon the reversal of precipitation regimes. Liu et al. [33] reported that nitrogen deposition generally reduces microbial biomass and diversity, yet increased precipitation can mitigate such adverse effects. Other studies have indicated that reductions in precipitation lead to decreased taxonomic diversity of microbial communities, whereas functional diversity exhibits divergent trajectories under the same conditions [34]. In the present study, however, precipitation variation did not significantly affect the alpha diversity of *nirK*-type denitrifying microorganisms—a finding that diverges from certain previous reports. Nevertheless, similar results have been documented under specific contexts. Dong et al. [35], investigating an arid year, also observed no significant influence of precipitation on microbial diversity, which is consistent with our conclusions. Studies have also revealed through redundancy analysis that soil water content is one of the key environmental factors driving the community structure of *nirK* and *nirS*-type denitrifying microorganisms [36]. Likewise, Li et al. [37], through a controlled precipitation manipulation experiment in northern Tibet spanning from −25% to +75%, found that while alpha diversity of soil microbial communities remained largely unaffected by precipitation changes, both community composition and species co-occurrence networks were substantially restructured. Furthermore, a study conducted across a low-precipitation gradient revealed that soil microbial abundance was negatively correlated with precipitation, yet microbial diversity remained insensitive to the precipitation gradient [38].

### 4.2. Effects of Precipitation Variations on Community Structure and Species Composition

Beta diversity analyses revealed that precipitation reduction was the primary driver of community structural divergence, while extreme wetting increased within-group heterogeneity. These patterns are consistent with previous work identifying mean annual precipitation as a strong predictor of *nirK* community composition: [39] identified mean annual precipitation (MAP) as a critical factor driving β-diversity changes in *nirK*-type denitrifier communities, accounting for 19% of the observed variation. Furthermore, while drought alone did not significantly alter the *nirK*/*nirS* gene abundance ratio, its interaction with nitrogen addition markedly increased the relative abundance of *nirK* over *nirS*. This indicates that precipitation changes, particularly drought, can distinctly shift the relative composition of the two major denitrifying microbial groups when coupled with other environmental stressors like nitrogen deposition, reflecting potential β-diversity alterations [40]. In alpine grassland ecosystems [41], simultaneous monitoring of gene abundances such as *nirS* and *nirK* revealed that nitrogen addition is the primary factor stimulating N_2_O emissions, while precipitation changes exerted no significant influence—a finding consistent with the conclusions of this study.

At the phylum level, Proteobacteria remained overwhelmingly dominant across all treatments, while at the genus level, *Bradyrhizobium* constituted the predominant taxon. Notably, *Ochrobactrum* increased significantly under reduced precipitation, whereas *Rhodopseudomonas* exhibited a higher relative abundance under increased precipitation. These patterns align with previous findings. For instance, a study on denitrifying communities in paddy soils reported that Proteobacteria was the dominant phylum within the *nirS*-type denitrifier gene pool, displaying the highest relative abundance [38]. Similarly, research on marine microbial communities demonstrated that Proteobacteria predominated in both *nirK*- and *nirS*-type denitrifying assemblages [42]. Saarenheimo et al. [43] further revealed that all operational taxonomic units (OTUs) of *nirK*-type denitrifying communities were affiliated with the genus *Ochrobactrum* within the Alphaproteobacteria, underscoring the pivotal role of Proteobacteria in *nirK*-mediated denitrification. The functional redundancy of *nirK* and *nirS* genes within *Bradyrhizobium* has also been documented, highlighting its ecological significance in denitrification processes [44]. Moreover, Zhang et al. [30] in a study of *nirS*-type denitrifiers in Qinghai Lake, similarly identified *Bradyrhizobium* as a principal bacterial genus contributing to denitrification. Wertz et al. [45] suggested that bacteria belonging to the genus *Rhodopseudomonas* may constitute key denitrifying taxa. Additionally, soil moisture content has been shown to exhibit a significant positive correlation with *nirK* gene abundance [20], implying that elevated humidity enhances the overall population size of *nirK*-type denitrifiers—a mechanism that may account for the increased abundance of *Rhodopseudomonas* observed under enhanced precipitation in the present study. In this study, Planctomycetes was significantly suppressed under extreme precipitation reduction. This finding is corroborated by a study on forest soil bacterial communities [46], which reported that Planctomycetes is highly sensitive to fluctuations in soil moisture content, suggesting its potential utility as a drought-sensitive indicator phylum.

### 4.3. Environmental Factor Driving Mechanisms

The present study identified pH as the primary environmental factor shaping both the diversity and community structure of *nirK*-type denitrifying microorganisms, a finding consistent with the well-established role of pH in structuring denitrifying communities [47]. For instance, Kou et al. [39] demonstrated that community assembly of *nirK*-type denitrifiers is driven by multiple edaphic variables—including soil pH, organic carbon, total nitrogen, carbon-to-nitrogen ratio (CNR), and plant diversity—and that pH exerts a stronger direct influence on *nirK* communities than do climatic factors. Furthermore, a separate investigation reported that the community structure of *nirK*-harboring denitrifiers was significantly correlated with soil pH, whereas no such association was detected for *nirS*-harboring communities with any measured environmental variable [48]. This distinction further underscores pH as a key deterministic filter governing the compositional differentiation of *nirK*-type denitrifying assemblages. In addition, we observed that temperature (Tem) and the CNR exerted substantial negative contributions to both the diversity and community structure of *nirK*-type denitrifiers. This negative contribution aligns with findings reported by Mooshammer et al. [49] and Pishgar et al. [50] who demonstrated that microbial nitrogen use efficiency is adaptively modulated in response to shifts in resource CNR stoichiometry as a strategy to mitigate carbon–nitrogen imbalance. The theoretical framework advanced by these authors posits that when the resource CNR becomes excessively high (i.e., under conditions of relative nitrogen scarcity), microbial communities enter a state of nitrogen limitation, prompting adjustments in both growth and metabolic strategies. This mechanistic interpretation directly supports the view that elevated CNRs constrain nitrogen-cycling processes in microbial communities. Similarly, previous research has documented that long-term warming imposes selective pressure on *nirK*-type denitrifying communities, leading to discernible shifts in community composition [51]. This finding offers a plausible explanation for the negative contribution of temperature observed in the present study, wherein sustained thermal stress may have differentially filtered *nirK* taxa, thereby restructuring community architecture.

### 4.4. Regulation of Microbial Functional Groups by Precipitation Variations

The opposing functional trajectories we observed—enhanced nitrogen fixation potential under drought versus amplified nitrate respiration under wetting—are ecologically coherent. Reduced precipitation typically lowers soil nitrogen availability, conferring a competitive advantage to nitrogen-fixing taxa [52,53]. Conversely, elevated soil moisture under increased precipitation promotes anaerobic microsite formation, creating favorable conditions for nitrate respiration [54]. This interpretation is supported by studies showing that increased precipitation stimulates heterotrophic respiration, including denitrification [55,56]. Importantly, our functional predictions derived from FAPROTAX were congruent with observed taxonomic shifts: drought-enriched *Ochrobactrum* includes known nitrogen-fixing strains, while wetting-enriched *Rhodopseudomonas* comprises facultative denitrifiers capable of nitrate respiration. This congruence between taxonomy and function strengthens the inference that precipitation variation drives meaningful functional restructuring.

### 4.5. Variable Selection Under Deterministic Processes

Species interactions within ecosystems encompass both positive and negative relationships, as well as direct and indirect associations [49]. In the present study, precipitation variability exerted strong selective pressure in the form of “variable selection” by modulating soil abiotic conditions, thereby rendering deterministic processes the dominant mechanism governing community assembly. This finding is consistent with our parallel investigation [57], which demonstrated that deterministic processes likewise predominated in the assembly of denitrifying microbial communities in Qinghai Lake wetlands. Moreover, this perspective is corroborated by additional studies [39,58], which confirmed that variable selection constitutes the primary process driving compositional shifts in *nirK*-type denitrifying communities. Conversely, heterogeneous selection has been identified as a key local assembly process shaping the geographic distribution patterns of such communities [59]. Collectively, these lines of evidence establish variable selection as a critical abiotic filtering process that governs the assembly of denitrifying microbial communities under fluctuating precipitation regimes. By modulating inter-species interactions, this deterministic mechanism ultimately influences the nitrogen cycling functions of wetland ecosystems.

### 4.6. Limitations of This Study

Despite providing a systematic analysis of the *nirK*-type denitrifying community—from species composition and network interactions to functional predictions and assembly mechanisms—this study has several limitations. First, the absence of concurrent nitrogen oxide (N_2_O, NO) flux measurements prevents a direct, quantitative link between microbial community changes and greenhouse gas emissions. FAPROTAX predictions suggest that precipitation shifts may regulate metabolic potentials such as nitrate respiration and nitrogen fixation, but without gas flux data, these predicted functional changes cannot be validated at the ecosystem-process level. Second, denitrification rates were not directly measured. As a key indicator of gaseous nitrogen loss, this omission limits our ability to infer functional activity from community structure. The current conclusions therefore rely primarily on compositional data and gene abundance, without confirming whether precipitation treatments genuinely altered denitrification activity per unit of soil.

Third, the study did not employ nitrogen flux integration methods to estimate regional nitrogen budgets. Such integration, which temporally accumulates instantaneous gas emissions or transformation rates, is essential for assessing the cumulative impact of precipitation treatments on nitrogen cycling. Its absence constrains evaluation of the net effect of precipitation changes on ecosystem-scale nitrogen retention or loss.

In summary, future research should integrate simultaneous measurements of nitrogen oxide fluxes, denitrification rates, and flux-based modeling. This comprehensive approach would establish a complete evidence chain linking microbial communities, process rates, and gas fluxes, thereby providing a more robust understanding of the mechanisms regulating nitrogen cycling in alpine wetlands under changing precipitation regimes.

## 5. Conclusions

Through a manipulative precipitation experiment in the Qinghai–Tibet Plateau alpine wetland, we show that short-term precipitation changes (±25%, ±50%) did not alter the alpha diversity (richness and evenness) of soil *nirK*-type denitrifying communities, indicating resilience of taxonomic richness to moisture fluctuation. Precipitation treatments did, however, profoundly reshape community composition. Reduced precipitation drove directional structural divergence, whereas extreme wetting introduced marked uncertainty, reflected in elevated within-group heterogeneity. Compositionally, Proteobacteria retained phylum-level dominance, but strategic genus-level turnover occurred: *Ochrobactrum* enriched under drought, *Rhodopseudomonas* under wetting. Functional predictions revealed a concomitant redistribution of metabolic potential—drought steered the community toward a conservative nitrogen-retention strategy, while wetting exacerbated denitrification-dominated N loss.pH and Hum were identified as the key environmental mediators of these shifts. Community assembly was governed exclusively by variable selection, a deterministic process through which precipitation-induced edaphic heterogeneity acted as the fundamental selective filter shaping distinct *nirK*-type assemblages. Collectively, these findings provide critical insight into how precipitation variability regulates denitrification in high-altitude wetlands and establish an empirical foundation for understanding microbial nitrogen cycling in alpine ecosystems under ongoing climate change.

## Data Availability

The raw data have been uploaded to NCBI, and its BioProject is PRJNA1423670.

## Supplementary Materials

The following supporting information can be downloaded at: https://www.mdpi.com/article/10.3390/biology15060512/s1, Table S1-alpha; Table S2-env2; Table S3-functional table.

## Abbreviations

The following abbreviations are used in this manuscript:

N_2_O	Nitrous Oxide
TC	Total Carbon
TN	Total Nitrogen
βNTI	Beta Nearest Taxon Index
C/N	Carbon to Nitrogen Ratio
Hum	Soil humus
Tem	Soil temperature
